# Integration of Distinct Analysis Strategies Improves Tissue-Trait Association Identification

**DOI:** 10.3389/fgene.2022.798269

**Published:** 2022-03-29

**Authors:** Zhijian Yang, Wenzheng Xu, Ranran Zhai, Ting Li, Zheng Ning, Yudi Pawitan, Xia Shen

**Affiliations:** ^1^ Biostatistics Group, State Key Laboratory of Biocontrol, School of Life Sciences, Sun Yat-sen University, Guangzhou, China; ^2^ Center for Intelligent Medicine Research, Greater Bay Area Institute of Precision Medicine (Guangzhou), Fudan University, Guangzhou, China; ^3^ Department of Medical Epidemiology and Biostatistics, Karolinska Institutet, Stockholm, Sweden; ^4^ State Key Laboratory of Genetic Engineering, School of Life Sciences, Fudan University, Shanghai, China; ^5^ Centre for Global Health Research, Usher Institute, University of Edinburgh, Edinburgh, United Kingdom

**Keywords:** tissue-trait association, omics data integration, genome-wide association studies (GWAS), transcriptomics, tissue specificity, likelihood inference

## Abstract

Integrating genome-wide association studies (GWAS) with transcriptomic data, human complex traits and diseases have been linked to relevant tissues and cell types using different methods. However, different results from these methods generated confusion while no gold standard is currently accepted, making it difficult to evaluate the discoveries. Here, applying three methods on the same data source, we estimated the sensitivity and specificity of these methods in the absence of a gold standard. We established a more specific tissue-trait association atlas by combining the information captured by different methods. Our triangulation strategy improves the performance of existing methods in establishing tissue-trait associations. The results provide better etiological and functional insights for the tissues underlying different human complex traits and diseases.

## 1 Introduction

Establishing associations between human complex traits and tissues or cell types is essential in current genetics and biology. It provides useful etiological and functional insights for understanding the regulatory mechanisms underlying complex traits and diseases and subsequently directs the clinical research and even treatment. However, one can hardly answer such a question systematically by traditional experimental designs.

Genome-wide association studies (GWAS) produced a tremendous amount of summary association data describing the associations between millions of single-nucleotide polymorphisms (SNPs) and various phenotypes. On the other hand, RNA-sequencing (RNA-seq) technology has generated high-throughput gene expression data not only in different tissues (Lonsdale et al., 2013) but also at the single-cell level (Jaitin et al., 2014; Habib et al., 2017). With these resources, we now have a chance to link particular tissues or cell types to a complex trait by investigating the genes that have coherent effects at the expression level and on the trait. This serves as a general approach that tackles the topic via genome-wide architecture.

Several methods have been proposed in recent years, prioritizing trait-relevant tissues or cell types (Trynka et al., 2013; Slowikowski et al., 2014; Calderon et al., 2017; Ongen et al., 2017; Finucane et al., 2018; Shang et al., 2020; Zhu et al., 2021). Most of these methods try to uncover this problem by identifying genes that are specifically expressed in certain tissues or cell types and, at the same time, regulate the trait according to GWAS (Slowikowski et al., 2014; Calderon et al., 2017; Finucane et al., 2018; Shang et al., 2020). Genotype-Tissue Expression (GTEx) consortium used tissue-specific expression quantitative trait loci (eQTL) data instead of using gene expression data directly, trying to establish eQTL-based associations between tissues and traits (Ongen et al., 2017).

Regardless of these available methods, this is a typical discovery problem where a yes/no inference needs to be made for each test. Normally with the same null hypothesis, applying different methods to the same data produces similar answers as the statistical power is similar. However, for the tissue-trait association problem, the different methods developed are sometimes distinct not only in their setup but also in producing distinct discoveries. For example, Calderon et al. (2017) detected that total cholesterol (TC) was associated with liver (*p* = 2 × 10^–4^), small intestine (*p* = 0.01), spleen (*p* = 0.04), and adrenal gland (*p* = 0.05), supported by, e.g., the nutrient absorption function of the small intestine and the link between spleen and lipid metabolism (Fatouros et al., 1995; Ai et al., 2018). While in Ongen et al. (2017)’s study, the top 5 enriched tissues for TC were liver (*p* = 2.05 × 10^–13^), pancreas (*p* = 3.83 × 10^–13^), thyroid (*p* = 9.85 × 10^–13^), uterus (*p* = 1.23 × 10^–8^), and small intestine (*p* = 5.59, ×, 10^–9^). Most tissues’ *p*-values were lower than 0.05, but the spleen was ranked 19/44 and the adrenal gland 21/44. Their results were quite different from those by Cameron et al. (1974) but claimed to be supported by traditional medical evidence (Pucci et al., 2000). Who to trust? Intuitively we now seem to have more confidence in the cholesterol-liver and cholesterol-intestine associations. Different assumptions or setups may capture different biological natures, but it could also be limited power that makes their results not agree well. Thus, to see the general picture, we need to systematically evaluate the associations between complex traits and tissues, making use of these available distinct methods to gain more information. This requires assessing the operating characteristics of different methods, which is impossible to do conventionally as a gold standard for each tissue-trait association is mostly absent.

In fact, for three or more distinct methods testing the same set of null hypotheses, the sensitivity and specificity of each method can be estimated without any gold standard (Pepe and Janes, 2007), as long as the methods are *conditionally independent* (distinct enough, e.g., with distinct assumptions or modelling logic). Thus, based on the estimated operating characteristics, one can combine information captured by different kinds of methods testing tissue-trait associations, since none of the methods could capture the full information of the underlying biology.

Here, we aim to integrate the results from different methods to better investigate the tissue-trait association problem. We approach this by: 1) applying three distinct methods on the same set of tissue-trait pairs; 2) conducting maximum likelihood estimation of the sensitivity and specificity of each method in the absence of a gold standard; 3) subsequently combining the results from these methods to generate a more credible tissue-trait association atlas.

## 2 Materials and Methods

### 2.1 Estimation of Operating Characteristics and Prevalence

Let random variable *A*_*i*_ represent the unobservable true association status for the *i*-th pair of tissue and trait, where *A*_*i*_ = 1 represents associated, and *A*_*i*_ = 0 unassociated. In our model, *ρ* = *P* (*A*_*i*_ = 1) is the same for any given *i*, so without losing generality, we use *A* to denote *A*_*i*_. Given a particular set of significance thresholds for K binary tests of the status of *A*, we have *K* random variables *Y*_1_, … , *Y*_*K*_, for each of *i* = 1, … , *n* pairs of tissues and traits. Writing the true- and false-positive rates of the K binary tests as *ϕ*_*k*_ = *P*(*Y*_*ik*_ = 1|*A* = 1) and *ψ*_*k*_ = *P*(*Y*_*ik*_ = 1|*A* = 0), respectively, the unknown parameters are the prevalence of tissue-trait association *ρ* = *P* (*A* = 1) and *θ* = (*ϕ*_*k*_, *ψ*_*k*_), *k* = 1, … , *K*. With *K*⩾3 observed tests, *ρ* and *θ* can be estimated by maximizing the likelihood function

$$L\left(\theta ,\rho \right)=\overset{n}{\underset{i=1}{\prod }}\left\{\rho P_{\theta }\left(Y_{i1},\ldots ,Y_{iK}|A=1\right)+\left(1-\rho \right)P_{\theta }\left(Y_{i1},\ldots ,Y_{iK}|A=0\right)\right\}.$$ (1)

As the available degrees of freedom, 2^*K*^ − 1, is no less than the number of parameters, 2*K* + 1. Assuming conditional independence of the K binary tests, i.e., given A and the outcome of any test *Y*_*i*_, one cannot predict the outcomes of the other tests, we have

$$L\left(\theta ,\rho \right)=\overset{n}{\underset{i=1}{\prod }}\left\{\rho \overset{K}{\underset{k=1}{\prod }}P_{\theta }\left(Y_{ik}|A=1\right)+\left(1-\rho \right)\overset{K}{\underset{k=1}{\prod }}P_{\theta }\left(Y_{ik}|A=0\right)\right\}.$$ (2)

For various *p*-value thresholds for different tissue-trait association test methods, we used quasi-Newton method for optimization to solve the above maximum likelihood problem. We bootstrapped the observed binary data to assess the variation of the estimates. In this article, we repeated the bootstrap procedure for 99 times. Our implementation is publicly available as an R package (see *Code Availability*), which can assess three or more methods simultaneously. For the scenario of three methods, the maximum likelihood estimates (MLE) of the parameters can be derived analytically (Pepe and Janes, 2007) (see Supplementary Appendix).

For a particular *p*-value threshold, with the estimated operating characteristics, we used the estimated specificity $\psi_{k}^{\prime }=1-\psi_{k}$ as weights to highlight the methods with high specificity. We derived a tissue-trait association specificity score to evaluate the associations. The score sums up the estimated specificity of the methods that gave a positive signal to a particular tissue-trait association, divided by the sum of specificity across all the methods:

$$S_{i}=\frac{\sum_{k=1}^{K}\left(Y_{ik}\psi_{k}^{\prime }\right)}{\sum_{k=1}^{K}\psi_{k}^{\prime }}.$$ (3)

The *K* variables *Y*_*i*1_, … , *Y*_*iK*_, for each of *i* = 1, … , *n* pairs of tissues and traits, are the binary results across *K* methods under a particular significance threshold. The score is ranged from 0 to 1, and the higher the score, the higher confidence of the association.

As operating characteristics were estimated, the false discovery rate (FDR) for each method can be subsequently calculated as *ψ*_*k*_ (1 − *ρ*)/(*ψ*_*k*_ (1 − *ρ*) + *ϕ*_*k*_ *ρ*). Then, corresponding to *S*_*i*_, the FDR for the *i*-th tissue-trait pair could be calculated to quantify the confidence of the association:

$${\text{FDR}}_{i}=\frac{\underset{k}{\prod }\left[I\left(Y_{ik}=1\right)\psi_{k}\left(1-\rho \right)+I\left(Y_{ik}=0\right)\psi_{k}^{\prime }\left(1-\rho \right)\right]}{\underset{k}{\prod }\left[I\left(Y_{ik}=1\right)\psi_{k}\left(1-\rho \right)+I\left(Y_{ik}=0\right)\psi_{k}^{\prime }\left(1-\rho \right)\right]+\underset{k}{\prod }\left[I\left(Y_{ik}=1\right)\phi_{k}\rho +I\left(Y_{ik}=0\right)\phi_{k}^{\prime }\rho \right]},$$ (4)

where $\phi_{k}^{\prime }=1-\phi_{k}$ . With the calculated FDR_*i*_, the overall FDR across all the tissue-trait pairs could be assessed by

$$\frac{\sum_{i=1}^{n_{+}}\left(\omega_{i}{\text{FDR}}_{i}\right)}{\sum_{i=1}^{n_{+}}\omega_{i}},$$ (5)

where $\omega_{i}=\varphi^{-1}\left({\text{rank}}_{S_{i}>0}\left(S_{i}\right)\right)+1$ and *n*_+_ = *∑*_*i*_ *I*(*S*_*i*_ > 0), so that methods with high/low FDR are down-/up-weighted. *φ* ^−1^ (rank (⋅)) stands for standard inverse-Gaussian transformation. $\sum_{i=1}^{n_{+}}\omega_{i}$ is the number of significant discoveries of our combined results.

### 2.2 Simulation

We set the true prevalence *ρ* = *P* (*A* = 1) while *A* indicate the true association status between traits and tissues. For each method, we gave the number of tests *n* = 1,000, 10,000, 100,000 and assigned 0–1 values to *Y*_*i*_, *i* = 1, … , *n* representing the binary test results. We gave the true values of the true- and false-positive rates of the *K* = 3 binary tests, i.e., *ϕ*_*k*_ = *P*(*Y*_*ik*_ = 1|*A* = 1) and *ψ*_*k*_ = *P*(*Y*_*ik*_ = 1|*A* = 0), and calculated the theoretical probability of each binary results combination *P*(*Y*_*i*1_, … , *Y*_*iK*_), assuming conditional independence. We randomly produced the binary results *Y*_*ik*_ (*Y*_*ik*_ ∈ {0, 1}) of the *K* = 3 methods from the multinomial distribution with the calculated corresponding probabilities *P*(*Y*_*i*1_, … , *Y*_*iK*_). Then, based on the simulated binary data *Y*_*ik*_, we estimated the operating characteristics of the *K* = 3 methods *θ* = (*ϕ*_1_, *ψ*_1_, *ϕ*_2_, *ψ*_2_, *ϕ*_3_, *ψ*_3_) and the prevalence *ρ* using our maximum likelihood procedure above. We repeated the simulation for 100 times to assess the variation of our estimates.

### 2.3 Choice of Three Different Methods

Based on the modelling logic, we classified the methods reviewed by Zhu et al. (2021) into four different categories (Supplementary Table S1): 1) methods assessing genetic effects enrichment on tissue-specific gene expressions, including LDSC-SEG (Finucane et al., 2018), deTS (Pei et al., 2019), and SNPsea (Slowikowski et al., 2014); 2) methods assessing the tissue-specific eQTL effects on a complex trait, including NTCS (Ongen et al., 2017) and eQTLEnrich (Gamazon et al., 2018); 3) methods modeling the genetic variance component using the gene expression data, including RolyPoly (Calderon et al., 2017), IGREX (Cai et al., 2020), and RhoGE (Mancuso et al., 2017); and 4) methods assessing the genetic effects in tissue-specific gene-gene co-expression networks, i.e., CoCoNet (Shang et al., 2020). With these, we chose the LDSC-SEG procedure (we named as LDSC), the NTCS procedure (we named as eQTL), and RolyPoly to represent the three distinct types of methods for subsequent evaluation. The network-based CoCoNet method is currently limited to ranking tissues for each given trait instead of multiple traits (Zhu et al., 2021).

### 2.4 Real Data Analysis

We collected the GWAS summary statistics of 27 complex human traits and diseases from different consortia (see *Data Availability*). We used tissue-specific transcriptome data from the GTEx project version 7. Samples were collected from 48 non-diseased tissue sites ( >70 samples for each tissue) across more than 700 individuals. The gene expression data between GTEx project version 6p and 7 are consistent (Supplementary Figure S7).

We applied RolyPoly (Calderon et al., 2017) and stratified linkage disequilibrium score regression (LDSC) (Finucane et al., 2018) on these data set to detect associations between each pair of the 27 traits and 48 tissues. See below for the pipeline details of these two methods. For the third eQTL method, we directly took the *p*-values from the report by Ongen et al. (2017) for the tissue-trait pairs. Ongen et al. analyzed the tissue-specific eQTL data from GTEx version 6p, which only covered 44 tissues. Thus, we abandoned the *p*-values of the four tissues missed by Ongen et al. from RolyPoly and LDSC results and the tissue-trait pairs with missing values. *p*-values of the same 1,008 tissue-trait pairs from these three distinct methods were passed onto our operating characteristics assessment procedure. We gave a particular *p*-value threshold to turn the *p*-values into binary status. Varying the *p*-value threshold, one can consider the estimated operating characteristics as a receiver operating characteristic (ROC) curve.

### 2.5 Stratified LD Score Regression Analysis

For every GWAS summary-level data, we performed LDSC to test heritability enrichment on tissue-specifically expressed genes in order to infer the associations between tissues and complex traits. We analyzed the tissue-level GTEx v7 gene expression data.

For GTEx data, we used the median TPM value across individuals to represent the gene expression in each tissue. In each gene expression data set, following the procedure by Skene et al. (2018), we calculated an expression specificity score for each gene, defined as the proportion of each gene’s expression in each tissue or cell type. Only protein-coding genes were kept for further analysis.

For every tissue or cell type, we selected the 10*%* of expressed genes with the highest expression specificity score as the specifically expressed gene set. Then we extracted the genome start and end positions of the genes in each specifically expressed gene set based on GRCh37 reference. In stratified LDSC analysis, SNPs within the specifically expressed genes were annotated as 1 otherwise 0.

LD scores for the annotated SNPs in each tissue and cell type were calculated based on a one centiMorgan (cM) window. Only HapMap3 SNPs were retained for the analysis. We included all the pre-built baseline annotations in the ldsc software as covariates in the stratified regression model. We used the -overlap-annot argument and frequency files 1000G_Phase3_frq.tgz to confine our analysis on SNPs with MAF >5% . The MHC region was excluded from our analysis by applying regression weights 1000G_Phase3_weights_hm3_no_MHC.tgz. Enrichment *p*-values (one-sided) were used to test associations between each tissue/cell type and a phenotype.

### 2.6 RolyPoly Analysis

RolyPoly tries to catch the relationship between the variance of GWAS-estimated SNP effects and gene expressions in a tissue or a cell type in order to prioritize trait-relevant tissues or cell types.

Following the RolyPoly package tutorial, first, each GWAS data file was formatted. The positions of SNPs match GRCh37 reference genome. Second, we labeled genes by ENSG IDs and filtered out non-protein-coding genes. For normalization, we transformed, across all genes, the expression values to their quantiles. Across all the tissues, we inverse-normal transformed the values to standard normal distribution quantiles. We took absolute values to ensure the expression values are positive as the tutorial recommends. Third, an annotation file of the genes is required, consisting of the chromosome, start and end of the block, and a block label. The block label corresponds to the gene IDs in the expression data set. Following the settings in the tutorial, we chose a 10 kb window as a block centered at each gene’s transcription starting site (TSS). We produced this annotation file from the Ensembl GRCh37 gtf file. Autosomal genes and SNPs were analyzed. For LD reference data, we used the files provided by the RolyPoly package, calculated via PLINK based on 1,000 Genomes phase 3 reference, filtered with *R* ^2^ > 0.2.

## 3 Results

### 3.1 Rationale of Sensitivity and Specificity Estimation Without a Gold Standard

Since the true biological relevance between a pair of tissue and trait is mostly unclear, it is challenging to evaluate the each method’s performance in detecting tissue-trait associations. To take advantage of different analysis strategies, we start by estimating each method’s operating characteristics in the absence of a gold standard. Under a particular *p*-value threshold, each method is a binary test on *n* pairs of tissues and traits. *Y*_*ik*_ represents the outcome of the *i*-th test using the *k*-th method, *k* = 1, 2, … , *K*. 2*K* + 1 parameters are unknown and to be estimated, including the sensitivity and specificity of each method (denoted jointly as *θ*) and the prevalence of true associations (denoted as *ρ*) in the *n* tissue-trait combinations. The likelihood function is

$$L\left(\theta ,\rho \right)=\overset{n}{\underset{i=1}{\prod }}\left\{\rho P_{\theta }\left(Y_{i1},\ldots ,Y_{iK}|A=1\right)+\left(1-\rho \right)P_{\theta }\left(Y_{i1},\ldots ,Y_{iK}|A=0\right)\right\}.$$ (6)

If only two methods are considered, the parameters in the likelihood function are unidentifiable, which is why we usually need a gold standard to compare two methods. However, when *K*⩾3, the available degrees of freedom (2^*K*^ − 1) becomes no less than the numbers of parameters (2*K* + 1) so that we can estimate *θ* and *ρ*. An uncheckable but usually justifiable assumption in practice is that the evaluated methods are *conditionally* independent, 15 meaning that for a single test, one cannot easily predict the result from one method based on the result from another. The assumption fits the scientific problem we face here. We implemented a maximum likelihood estimation procedure to estimate *θ* for any number of *K* (see *Code Availability*).

### 3.2 Simulation

**FIGURE 1:**
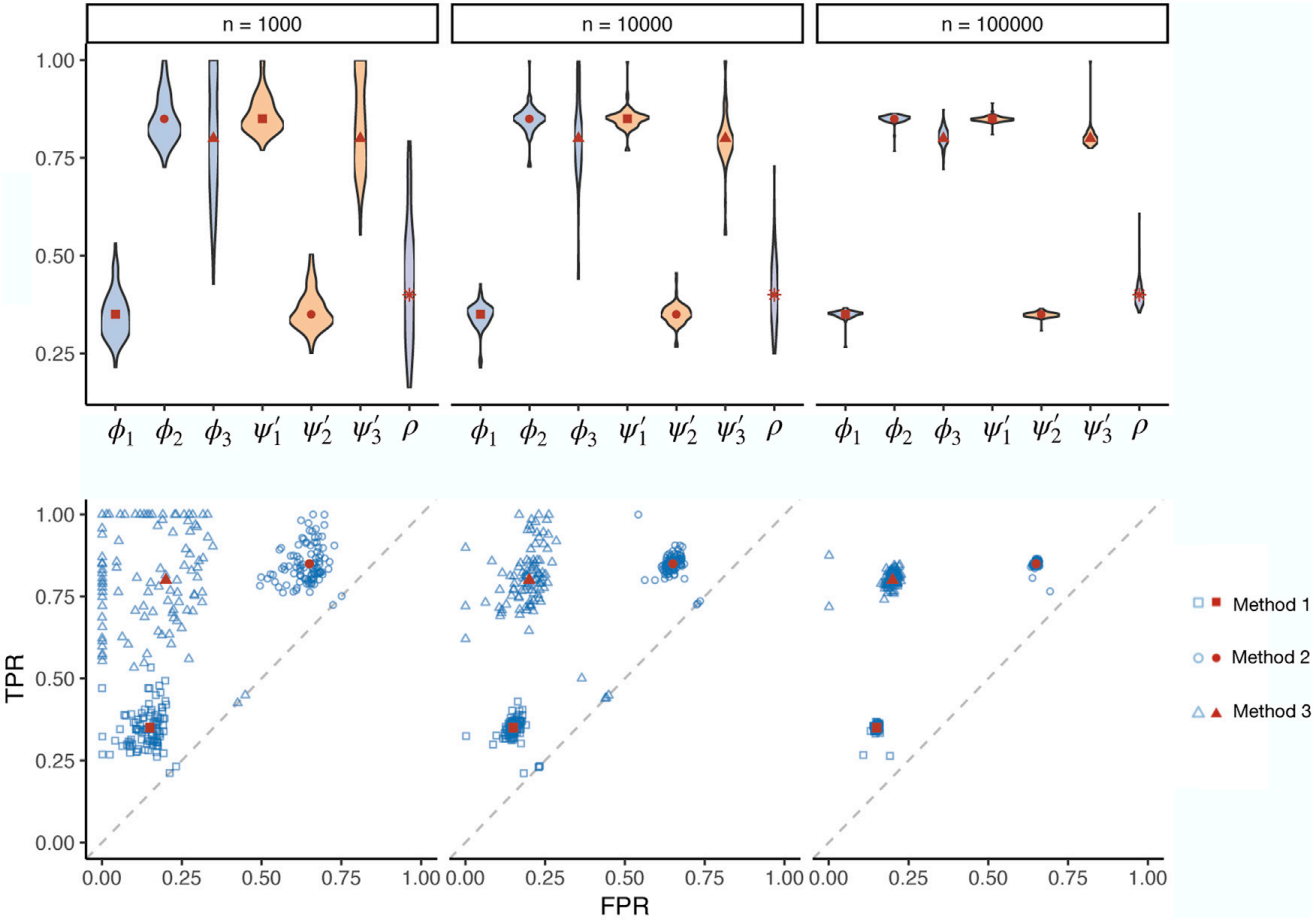
Simulations assessing the maximum likelihood estimation of operating characteristics in the absence of a gold standard. Three methods (represented by squares, circles, and triangles, respectively) with known true sensitivities and specificities (red dots) were simulated, applied on different numbers of tests in total (*n*) with a pre-defined prevalence of true positives. The estimated (blue dots) prevalence (*ρ*), sensitivity (*ϕ*), and specificity (*ψ*′) parameters are visualized for 100 simulation repeats. TPR: true positive rate. FPR: false positive rate.

Different method uses different modelling logic and even data sources. As the true biological relevance between a tissue and a trait can hardly be simulated without any bias that favours a particular method, a gold standard cannot be simulated to examine different methods. Nevertheless, simple true-false association status across a set of tissue-trait pairs can be simulated, useful for testing the validity of our maximum likelihood estimation procedure. We simulated three conditionally independent methods with pre-defined prevalence, sensitivity, and specificity values. The pre-defined specificities of the three methods were 0.85, 0.35, 0.8, and the sensitivities were 0.35, 0.85, 0.8. With the simulated binary test results, we estimated the operating characteristics of the three methods and prevalence (Figure 1, Supplementary Figure S2). The sample size *n* is the number of tests by each method in one simulation. We repeated the simulation 100 times for each simulation setting to obtain the empirical distribution of the estimates. The receiver operating characteristic (ROC) results showed that our estimates were consistent. The estimates converged to the true values as the sample size increased. We also tested our procedure under different levels of true prevalence (Supplementary Figures S3,S4). The estimation efficiency decreased when the prevalence value was close to the boundary of the parameter space.

### 3.3 Evaluation of Tissue-Trait Associations

We summarized the similarities and differences between three methods testing for tissue-trait or cell-type-trait associations (Supplementary Table S1). These three methods were so different, based on distinct theories and assumptions, that they could be considered conditionally independent–For each test between a tissue and a trait, given the result from one method, one can hardly predict the result from another method.

The first method RolyPoly is a hierarchical polygenic model to evaluate the association between gene-level variant effects and gene expression (Calderon et al., 2017). The second method has been particularly applied to link brain cell types to schizophrenia and Parkinson’s disease (Skene et al., 2018; Bryois et al., 2020). It uses stratified linkage disequilibrium score regression (LDSC) (Finucane et al., 2018) to test whether the SNP heritability of a trait is enriched on the specifically expressed genes in a given tissue or cell type. We applied LDSC using the same setup as described by Skene et al. (2018) The third method was conducted by the Genotype-Tissue Expression (GTEx) consortium, which used cis-eQTL effects data to infer causal tissues for complex traits by implementing the regulatory trait concordance (RTC) score (Nica et al., 2010). We named it eQTL in this paper and directly extracted the *p*-values reported by the GTEx consortium (Ongen et al., 2017).

We preprocessed publicly available GWAS summary statistics of 27 complex traits and diseases from different global consortia (see *Data Availability*). Based on these same GWAS summary statistics and the same gene expression data of 48 tissues from the GTEx project, we obtained tissue-by-trait *p*-value matrices for RolyPoly and LDSC, respectively. We extracted *p*-values of the eQTL method for the same GWASed traits from the original publication by the GTEx consortium (Ongen et al., 2017), which covered 44 tissues from GTEx project V6p. Removing missing values from the results, we obtained a total of 1,008 tissue-trait association tests of each method for further estimation (Supplementary Table S2, Supplementary Figures S1, S5). Under each particular *p*-value threshold, the eQTL method always produced more positive results, and RolyPoly always produced less.

**FIGURE 2:**
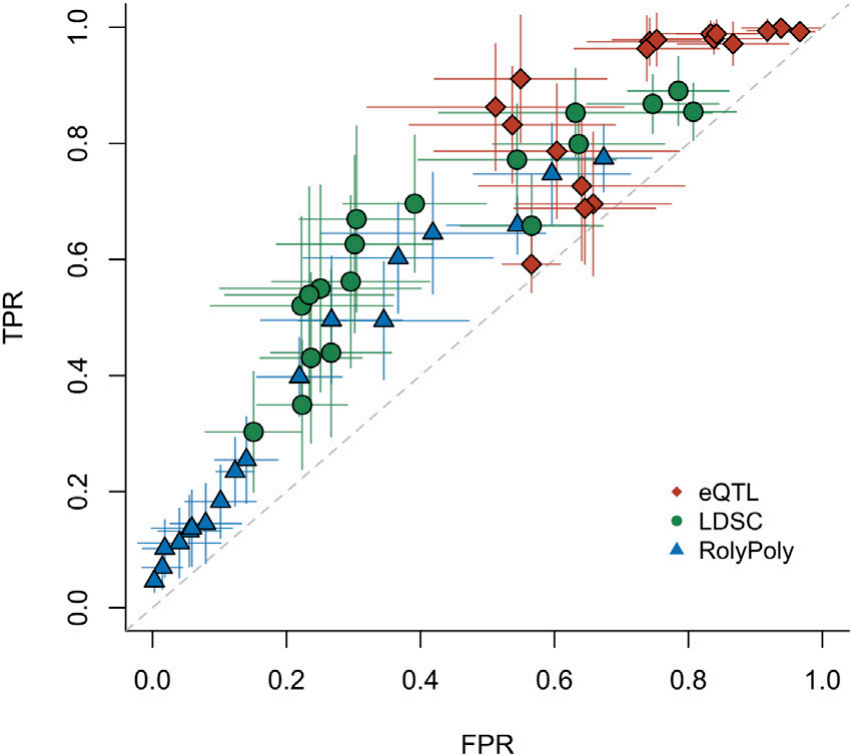
ROC for three distinct methods. For the real data application testing 27 traits v.s. 44 tissues, we estimated the operating characteristics of each method (eQTL, LDSC, and RolyPoly). Each point represents the mean of 99 bootstrap estimates. The whiskers give the standard errors of TPRs and FPRs based on the bootstrap estimates. Each point was evaluated under a particular *p*-value threshold: 0.01, 0.02, … , 0.09, 0.1, 0.2, … , 0.9. TPR: true positive rate. FPR: false positive rate.

We estimated the three methods’ operating characteristics via maximum likelihood under different *p*-value thresholds. We bootstrapped the binary test results of the three methods and performed the estimation procedure 99 times to obtain standard errors of the operating characteristics (Figure 2, Supplementary Table S3, Supplementary Figure S6). All three methods showed limited statistical power in detecting tissue-trait associations. The results also indicated that the small *p*-values produced by the eQTL method were likely inflated, which led to low specificity and generated many positive results that could not be rediscovered by any other method. The method RolyPoly discovered much less positive results but meanwhile had higher specificity. In general, the stratified LDSC method had a slightly better sensitivity-specificity trade-off than the other two methods.

### 3.4 A Combined Tissue-Trait Association Atlas

We defined an association score to assess the association between each pair of tissue and complex trait, i.e., the sum of the estimated specificity values of the methods that reported a positive association, standardized to range from 0 (no association) to 1 (highest strength). Conditioned on the positive discoveries, we incorporated the false discovery rate (FDR) of each method to calculate the FDRs for the combined association scores (see Methods for technical details).

**FIGURE 3:**
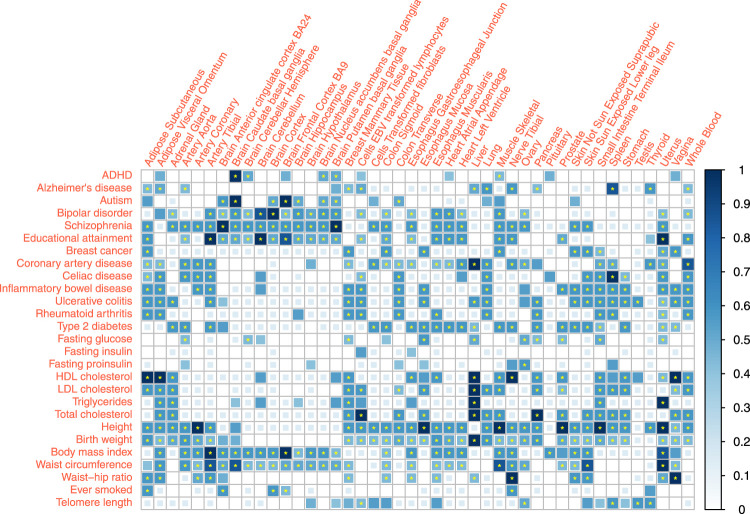
Tissue-trait association scoring combining specificity estimates of three distinct methods. Association between 27 traits and 44 tissues were quantified by association score. The association score sums the binary results from different methods, weighted by the specificity estimates. The *p*-value thresholds are 0.05 for all three methods (eQTL, LDSC, and RolyPoly). * represent < 0.05 for the combined false discovery rate (FDR) of the association score.

We reported the combined association score for the associations between 27 traits and 44 tissues under a 0.05 *p*-value threshold and evaluated the corresponding FDR (Figure 3, Supplementary Table S4). 297 out of the 1,188 trait-tissue pairs scored 0, indicating no evidence of an association. Among the remaining 891 pairs with non-zero scores, 421 association scores had a combined FDR <0.05 .

Many complex traits or diseases have associations with different tissues all over the body except brain tissues, such as coronary artery disease (CAD), height, and birth weight. It reflected the biological complexity of those phenotypes. In particular, blood lipids showed strong signals in the liver and also associations with the adrenal gland, visceral adipose tissue (VAT), small intestine, and whole blood. Conversion of blood cholesterol into cortisol hormone in the adrenal (Borkowski et al., 1967) and adipose metabolism function of VAT supported these associations. Type 2 diabetes (T2D) had strong signals on the pancreas, aorta, and gastrointestinal tissues. Immune diseases, including rheumatoid arthritis (RA) and gastrointestinal immune diseases, showed high consistency, associated with the spleen (Fishman and Isenberg, 1997; Di Sabatino et al., 2005; Sabatino et al., 2013), lung, EBV (Epstein-Barr virus) transformed lymphocytes, whole blood (Pauley et al., 2008), adipose tissues, and small intestine (terminal ileum). Different from RA, gastrointestinal immune diseases were also associated with stomach (Büning et al., 2015). The profiles of gastrointestinal immune diseases were similar.

Educational attainment and most psychiatric disorders showed robust brain-specific associations. The link between spleen and Alzheimer’s disease (AD) indicates that AD is immune-related and is not significantly related to the brain tissues. This finding agrees with the theory that immunity and inflammation play an essential role in AD (Heppner et al., 2015).

Fasting phenotypes showed associations with fewer tissues. Fasting glucose level was significantly linked to the pancreas, the same as T2D. However, the pancreas association was not significant for fasting insulin and proinsulin levels, possibly due to the limited statistical power of these GWAS and low heritability of these two traits.

Among the obesity-related anthropometric traits, only the waist-hip ratio (WHR) was strongly associated with subcutaneous adipose tissue. Such an association cannot be detected in body mass index (BMI) and waist circumference, regardless of the large sample size of BMI GWAS meta-analysis. Such results suggest WHR as a more robust quantity that captures adipose-induced obesity. BMI and waist circumference may be more easily influenced by the postnatal environment or regulated by nerves or hormones while showing more associations with the brain. We noted that obesity measurements did not share a similar profile with blood lipids.

Uterus showed strong associations with traits like Educational attainment, birth weight, and breast cancer. Ovary also showed no or weaker associations with these traits. However, other genital organs, such as the testis and vagina, had no similar signals. These findings likely implied the maternal genetic effects on these traits, potentially relevant to pregnancy (Pickett et al., 2000).

### 3.5 Incorporating Multiple Methods Resulted in Better Discovery Performance

**FIGURE 4:**
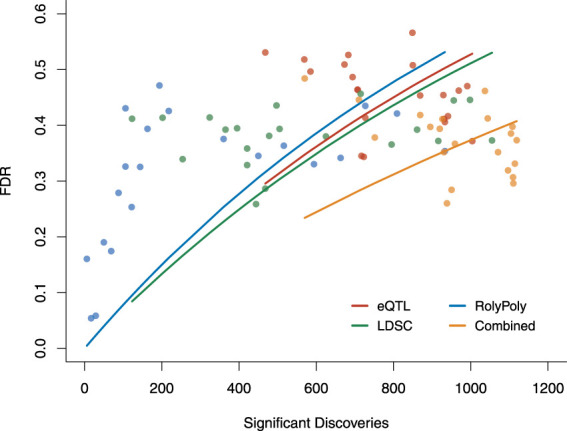
The overall performance of three methods and the combined results in terms of FDR v.s. the number of claimed significant discoveries. We calculated the FDR for each method and the overall FDR for the combined result based on the estimated operating characteristics. Each point was evaluated under a particular *p*-value threshold: 0.01, 0.02, … , 0.09, 0.1, 0.2, … , 0.9. Each curve was fitted for the exponential model of *Y* = 1 − *e* ^−*aX*^. FDR: false discovery rate.

Based on the three methods’ estimated operating characteristics, analog to standard ROC, we examined each method’s FDR as a function of its number of claimed positives (Figure 4, Supplementary Table S5). Though the LDSC procedure showed the best overall performance for detecting tissue-trait associations, the performance of the three methods was similar and limited. Above, we constructed an association score combining the results claimed by different methods, where we re-weighted the three methods based on their specificities. This led to better performance. The combined FDR was lower than each method, and we had more significant discoveries than each method at the same FDR level.

## 4 Discussion

In the absence of a gold standard, we assessed the operating characteristics of three methods that prioritize tissues associated with complex traits and diseases. Then, we evaluated the estimation procedure in simulation and obtained a ensemble-analyzed score to construct tissue-trait associations by integrating results from different methods. We proved that our results had better FDR level than results from other existing methods. With practice, we established a more credible association atlas across 44 human tissues and 27 complex traits and diseases.

Our analysis is different from conventional meta-analysis. Normally, meta-analysis refers to researchers combining results obtained from different samples/studies using the same method (or very similar methods), in order to reach larger sample size thus higher statistical power. However, we combined different results from distinct methods based on the same/similar data source. This is similar to the “ensemble” idea in the field of machine learning. Thus, we are not performing any meta-analysis for power (to improve sensitivity) but rather gathering more information to reduce FDR (to improve specificity).

Zhu et al. (2021) reviewed the existing methods, we still lack a systematic evaluation of them with a quantified estimate on performance. We emphasize that our estimation of the sensitivity and specificity of each method requires the assumption that the methods testing the same null hypothesis are *conditionally independent*. This means that the methods considered in our likelihood inference need to be as distinct as possible, preferably based on distinct assumptions and modelling logic. Therefore, simultaneously including similar methods based on similar modeling ideas would obviously violate the conditional independence assumption. The three methods we chose differ in the underlying statistical models, and they indeed produce sufficiently distinct results to justify conditional independence. Our assessment of the operating characteristics found the limited performance of the RolyPoly and eQTL-based methods. LDSC, focusing on the enrichment of heritability or genetic association signals on tissue-specific gene expressions, showed slightly better performance. Such an observation indicates that methods that properly account for LD structure across the genome tend to have less noise in the inferential process. The eQTL method appeared to have inflated sensitivity as it does not consider genome-wide architecture.

When we assess three imperfect testing methods, while no gold standard exists, the assumption of our likelihood inference is that the methods are *conditionally independent*. Under this assumption, if two methods report similar results, then we have more confidence on such results, as we have evidence from two conditionally independent sources. Nevertheless, it is still possible that a good method might be down-weighted by two bad/biased methods, especially when the two bad methods are too much underpowered, i.e., simultaneously reporting a large number of negatives. So, in practice, we need to consider methods that have reasonable statistical power, so that our combined inference results can gain more information and produce more specific discoveries with smaller FDR.

Although the sensitivity and specificity parameters are the key in our analysis, the prevalence parameter *ρ* might also be of interest, referring to the global proportion of true associations. The interpretation of *ρ* would be more straightforward if we could estimate a trait-specific proportion of truly associated tissues; However, this would only be ideal if the number of tissues assessed is large. In the current analysis, having only an overall prevalence parameter would maximize our power for estimating the key sensitivity and specificity parameters. The estimated prevalence only slightly varies as long as the thresholds for the three testing methods are not too extreme (Supplementary Table S3).

The methods for establishing tissue-trait associations generally link genome-wide association signals to tissue-specific gene expression information. If we look into the genetic correlations (Zheng et al., 2017) (Supplementary Table S6), it is not surprising that similarities of tissue-association profiles between the traits appeared to be positively correlated with their genetic correlations. For example, bipolar disorder and schizophrenia had a high genetic correlation (*r*_*g*_ = 0.832, *p* = 5.36 × 10^–106^). However, some trait pairs with high genetic correlation didn’t quite share a similar tissue-association pattern, such as waist circumference and waist-hip ratio (*r*_*g*_ = 0.733, *p* = 0), and BMI and waist-hip ratio (*r*_*g*_ = 0.547, *p* = 1.79, ×, 10^–65^). Though we usually consider these three traits as similar predictors of health, it seems that waist-hip ratio might be affected by some particular pathways in the subcutaneous adipose tissue rather than in brain tissues or neurons. The trait pairs with high genetic correlations but not so similar tissue-association profiles may suggest new insights on tissue biology and underlying pathways.

Compared to previous studies that applied single methods, we obtained more specific trait-relevant tissues. Nevertheless, we still lack clear biological explanations for many tissue-trait associations. For example, there is a link between human height and artery: Does it reflect the negative genetic correlation between heart disease and height (Nelson et al., 2015), or does it imply any function of the artery in growth? The tissue-trait associations gave us new knowledge for the genetic architecture of complex traits, but more community efforts are needed to explore the underlying physiological and biological connections between complex traits and tissues or cell types. The association atlas established here helps understand the regulatory mechanisms underlying complex traits and may assist experimental designs and potential clinical research in the future.

## 5 Code Availability

The code we used in our analysis is available as an R package triangulation at: https://github.com/xiashen/triangulation. The software LDSC we used in this paper is available at https://github.com/bulik/ldsc. The R package RolyPoly for inferring relevant cell types and tissues for complex traits is available at https://github.com/dcalderon/rolypoly.

## Data Availability

The original contributions presented in the study are included in the article/Supplementary Material, further inquiries can be directed to the corresponding author.
