# Supplementary material for: Integration of Distinct Analysis Strategies Improves Tissue-Trait Association Identification
Source: Front Genet. 2022 Mar 29;13:798269. doi: 10.3389/fgene.2022.798269 (PMC9014299; doi:10.3389/fgene.2022.798269)
Supplement: Supplementary file 1 [file Presentation1.pdf]

Supplementary Material

for

Integration of distinct analysis strategies improves tissue-trait association identification

by Zhijian Yang, Wenzheng Xu, Ranran Zhai, Ting Li, Zheng Ning, Yudi Pawitan, Xia Shen

Appendix

Derivation of the maximum likelihood estimators for three methods

To more intuitively understand how the raw frequencies are used to obtain the estimated operating characteristics via maximum likelihood, here we re-derive the analytic form of maximum likelihood estimators. The derivation corrects a few errors in latent class analysis literature (Pepe and Janes, 2007). As we define in the Method section, we consider $K = 3$ imperfect binary tests for tissue-trait associations and the testing binary results $Y = \{Y_{11}, ..., Y_{ik}, ..., Y_{n3}\}$ for n samples, $k = 1, 2, 3$. When $K = 3$, we wrote the probabilities of observation as $p_k = P(Y_k = 1), k = 1, 2, 3$; $p_{kj} = P(Y_k = 1, Y_j = 1), j > k, j \in (2, 3)$; and $p_{123} = P(Y_1 = Y_2 = Y_3 = 1)$. The seven unknown parameters are $(\rho, \theta) = (\rho, \{(\varphi_k, \psi_k), k = 1, 2, 3\})$, i.e., the prevalence of true associations and the sensitivity and specificity of each method. If three methods are conditionally independent, we have the seven equations below about the observational probabilities:

$$p_k = \rho\varphi_k + (1-\rho)\psi_k,\ k = 1, 2, 3 \tag{A.1}$$

$$p_{kj} = \rho\varphi_k\varphi_j + (1-\rho)\psi_k\psi_j,\ k < j,\ j \in (2,3) \tag{A.2}$$

$$p_{123} = \rho\varphi_1\varphi_2\varphi_3 + (1-\rho)\psi_1\psi_2\psi_3 \tag{A.3}$$

Denote ψ_k using (P, ρ, φ_k) according to (A.1),

$$\psi_k = (p_k - \rho\varphi_k)/(1-\rho),\ k \in (1, 2, 3) \tag{A.4}$$

and then substitute into (A.2) to yield

$$(p_k - \varphi_k)(p_j - \varphi_j) = \frac{1-\rho}{\rho}(p_{kj} - p_k p_j),\ k < j,\ j \in (2,3)$$

Thus, we have:

$$\varphi_k = p_k - \frac{1-\rho}{\rho}\frac{p_{1k} - p_1 p_k}{p_1 - \varphi_1},\ k = 2, 3,$$

and substituting into the above equation,

$$\begin{aligned}(p_1 - \varphi_1)^2 &= \frac{(p_{12} - p_1 p_2)(p_{13} - p_1 p_3)}{p_{23} - p_2 p_3}\frac{1-\rho}{\rho} \\ &= C_1(1-\rho)\rho,\end{aligned}$$

where

$$C_k = \frac{(p_{kj} - p_k p_j)(p_{kl} - p_k p_l)}{p_{jl} - p_j p_l}, \{j, k, l\} = \{1, 2, 3\}.$$

Thus,

$$\varphi_k = p_k \pm \sqrt{C_k}\sqrt{(1-\rho)/\rho}. \tag{A.5}$$

Substituting φ_k into (A.4), we obtain

$$\begin{aligned} \psi_k &= (p_k - \rho p_k \pm \sqrt{C_k}\sqrt{(1-\rho)\rho})/(1-\rho) \\ &= p_k \pm \sqrt{C_k}\sqrt{\rho/(1-\rho)}. \end{aligned} \tag{A.6}$$

There are two solutions for $\varphi_k : p_k \pm \sqrt{C_k}\sqrt{(1-\rho)/\rho}$. We choose $\varphi_k = p_k + \sqrt{C_k}\sqrt{(1-\rho)/\rho}$ which follows a reasonable assumption that the true positive rate is at least as large as the false positive rate, i.e., $\varphi_k \geq \psi_k$. So that $\psi_k = p_k - \sqrt{C_k}\sqrt{\rho/(1-\rho)}$.

Substituting expression fro φ_k and ψ_k into (A.3) yields

$$p_{123} = p_1p_2p_3 + p_2\sqrt{C_1C_3} + P_3\sqrt{C_1C_2} + p_1\sqrt{C_2C_3} + \sqrt{C_1C_2C_3}(\rho\sqrt{\rho/(1-\rho)} - \sqrt{(1-\rho)/\rho}(1-\rho)).$$

Note that

$$C_kC_j = (p_{kj} - p_kp_j)^2,$$

so defining

$$V = \frac{p_{123} - p_{12}p_3 - p_{13}p_2 - p_{23}p_1 + 2p_1p_2p_3}{\sqrt{(p_{12} - p_1p_2)(p_{13} - p_1p_3)(p_{23} - p_2p_3)}},$$

we have

$$\rho\sqrt{\rho/(1-\rho)} - \sqrt{(1-\rho)/\rho}(1-\rho) = V$$

$$\begin{aligned} V^2 &= \frac{\rho^3}{1-\rho} - 2\rho(1-\rho) + \frac{1 - 3\rho + 3\rho^2 - \rho^3}{\rho} \\ &= -4 - \frac{1}{\rho(1-\rho)}. \end{aligned}$$

Eventually,

$$\rho = \frac{1}{2} \pm \sqrt{\frac{1}{4} - \frac{1}{4 + V^2}}. \tag{A.7}$$

Insight into the estimators

For $K = 3$ testing methods and the observable probabilities $p_k = P(Y_k = 1), k = 1, 2, 3$; $p_{kj} = P(Y_k = 1, Y_j = 1), j > k, j \in (2, 3)$; and $p_{123} = P(Y_1 = 1, Y_2 = 1, Y_3 = 1)$. We have shown above that there is a one-to-one map from the observable probabilities to the seven parameters to be estimated. As the MLE of the observable probabilities are simply the corresponding data frequencies, we obtain the following analytic expressions for the MLE of the parameters, assuming conditional independence:

$$\widehat{\varphi_k} = \widehat{p_k} + \sqrt{\widehat{C_k}}\sqrt{(1 - \widehat{\rho})/\widehat{\rho}}, \tag{A.8}$$

$$\widehat{\psi_k} = \widehat{p_k} - \sqrt{\widehat{C_k}}\sqrt{\widehat{\rho}/(1 - \widehat{\rho})}, \tag{A.9}$$

where C_k as described above and

$$\widehat{\rho} = \frac{1}{2} \pm \sqrt{\frac{1}{4} - \frac{1}{4 + \widehat{V}^2}}, \tag{A.10}$$

where V as defined above. We choose the $\widehat{\rho}$ with the larger likelihood so that MLE are achieved.

These formulae describe how the observed frequencies from the raw data are used to infer the latent prevalence of true tissue-trait associations and the operating characteristics of the three methods. For the estimated ρ, the analytic expression (A.10) reveals that the starting point for the estimation is $\widehat{\rho} = 1/2$, with $\widehat{V}$ determining its deviation from 0.5. Given the estimated $\widehat{\rho}$, the performance of method k is determined by the factor C_k. C_k is a ratio, where the numerator reflects the agreement between method k and the other two methods, and the denominator indicates the agreement between the other two methods. Intuitively, if the results from method k can be confirmed by the other two methods, we have more confidence on its performance; on the other hand, if the other two methods report more consistent results, method k will be down-weighted in its performance.

Supplementary Tables

Table S1: Categorization of tissue-trait association methods according to the modelling logic. The three chosen methods for evaluation and combination in this paper are highlighted in bold.

Method category	Method name	Input data besides GWAS summary statistics	Test statistics	Statistical model
Modeling the genetic variance component using the gene expression data	**RolyPoly**	Gene expression data	p-values	A regression-based polygenic model
	IGREX	eQTL effects data	p-values	Linear mixed model via restricted maximum likelihood (REML)
	RhoGE	Gene expression data	p-values	Two-stage regression
Assessing genetic effects enrichment on tissue-specific gene expressions	**LDSC** (LDSC-SEG)	Gene expression data	p-values	LD score regression
	deTS	Gene expression data	p-values	Tissue-specific enrichment test for trait-relevant genes
	SNPsea	Gene expression data	p-values	Tissue-specific enrichment test for trait-relevant genes
Assessing the tissue-specific eQTL effects on complex traits	**eQTL** (NTCS)	eQTL effects data	Ranking of tissues based on adjusted fold-enrichment	Regulatory trait concordance (RTC)
	eQTLEnrich	eQTL effects data	p-values	Enrichment test
Assessing the genetic effects in tissue-specific gene-gene co-expression networks	CoCoNet	Gene co-expression network information	Ranking of tissues based on log-likelihood	Covariance regression network model

Table S2: P-values from the three methods analyzing associations between 27 traits and 44 tissues.

[See the Excel File]

Table S3: Estimated operating characteristics and prevalence under different p-value thresholds.

[See the Excel File]

Table S4: Association scores and combined FDRs under 0.05 thresholds.

[See the Excel File]

Table S5: Significant discoveries and FDR of each method under different thresholds.

[See the Excel File]

Table S6: Genetic correlations across 25 traits from LD Hub.

[See the Excel File]

Supplementary Figures

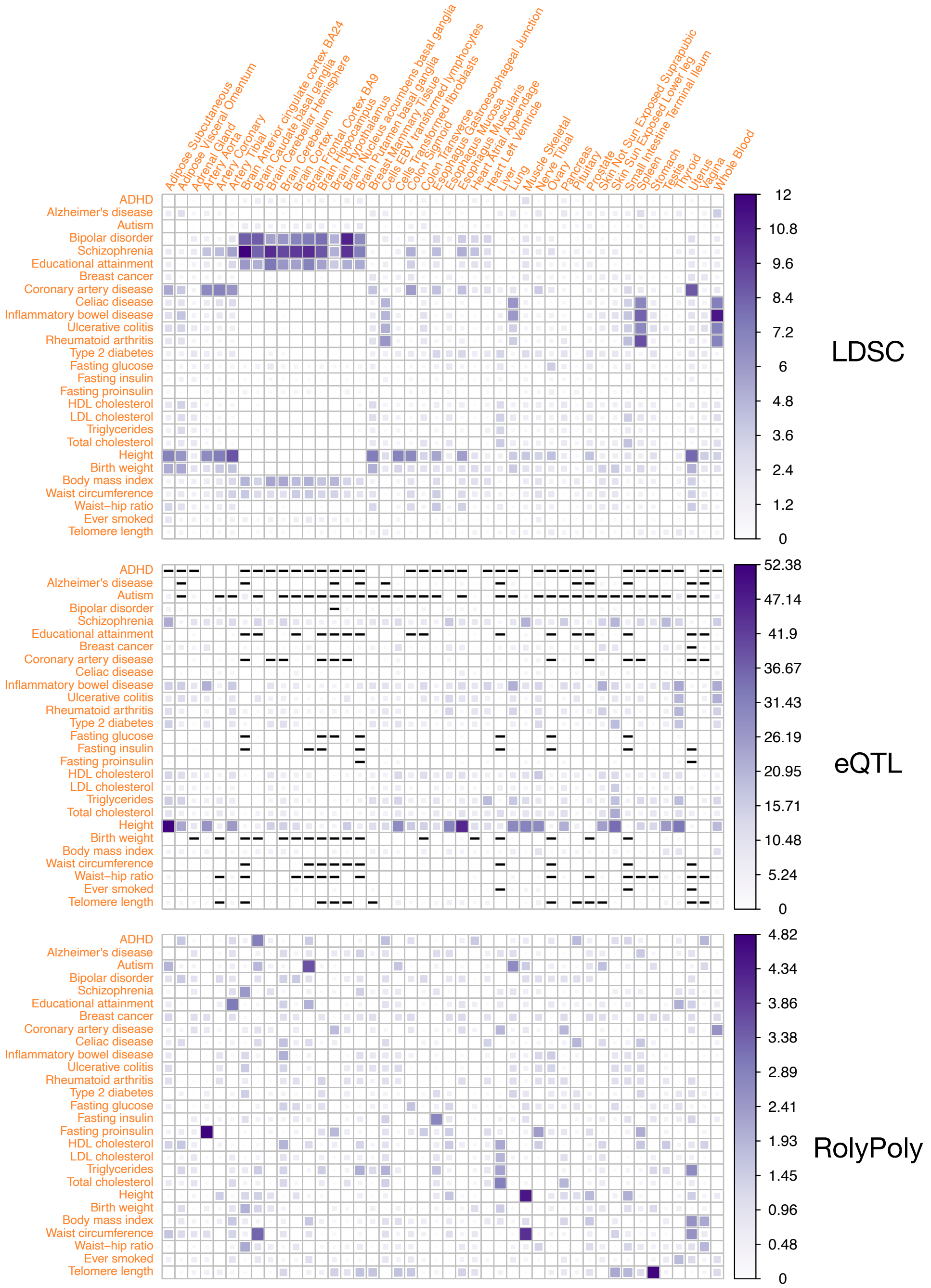

Figure S1: Heatmaps for the tissue-trait association $-\log_{10} P$-values by the three methods. The color key refers to $-\log_{10} P$-values produced by each method. The horizontal bars represent missing values.

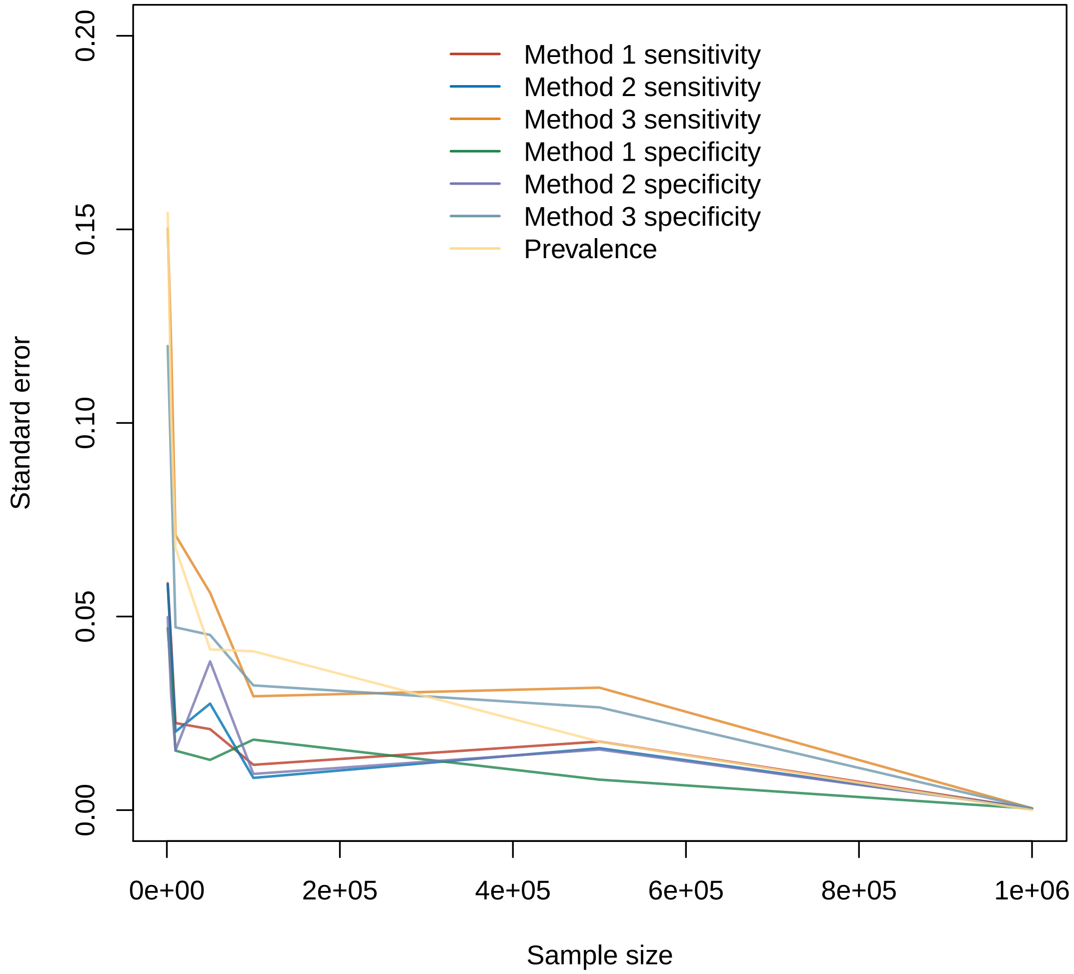

Figure S2: Relationship between simulated sample sizes and estimated standard errors. Each standard error was obtained from 99 times bootstrap resampling. The pre-defined true values of operating characteristics and prevalence were the same as in Figure 1.

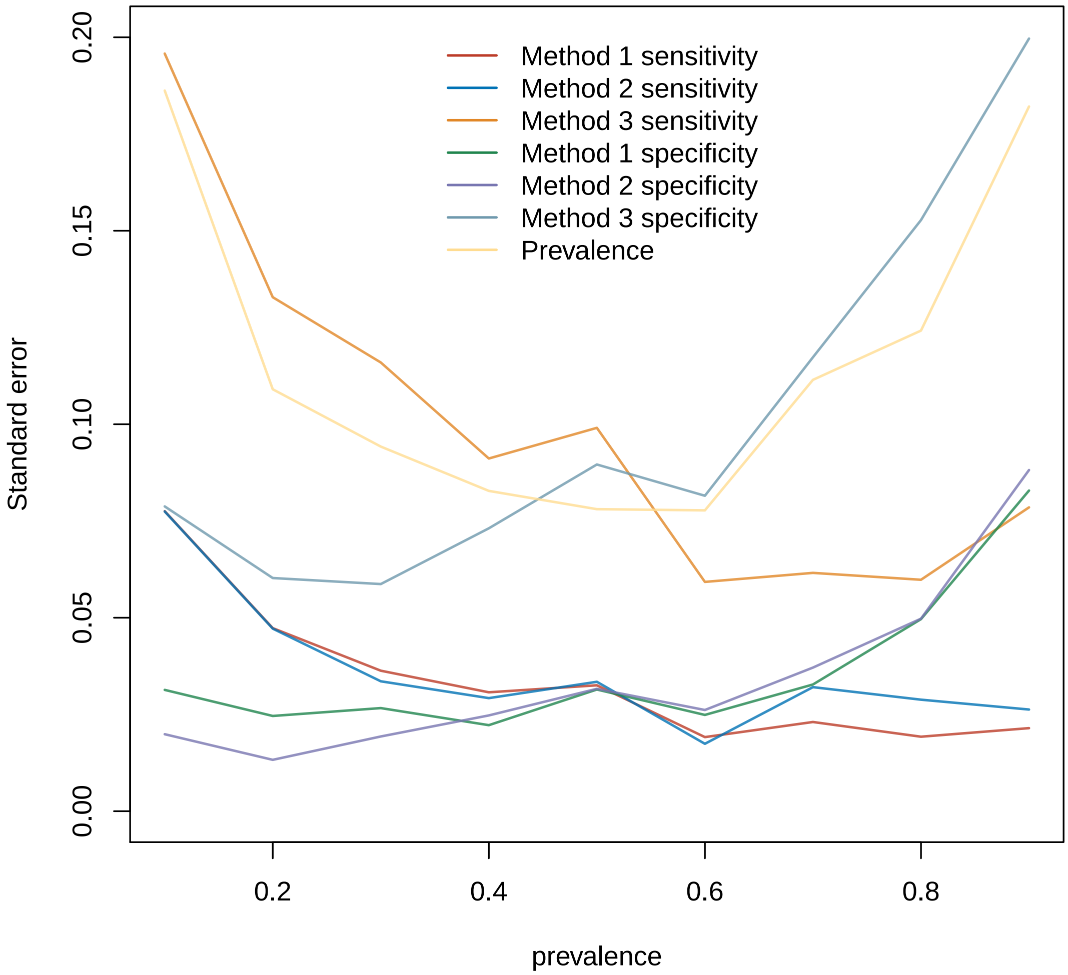

Figure S3: Relationship between simulated prevalence values and estimated standard errors. The simulated sample size was $10,000$. Each standard error was obtained from 99 times bootstrap resampling. The pre-defined true values of the operating characteristics were the same as in Figure 1.

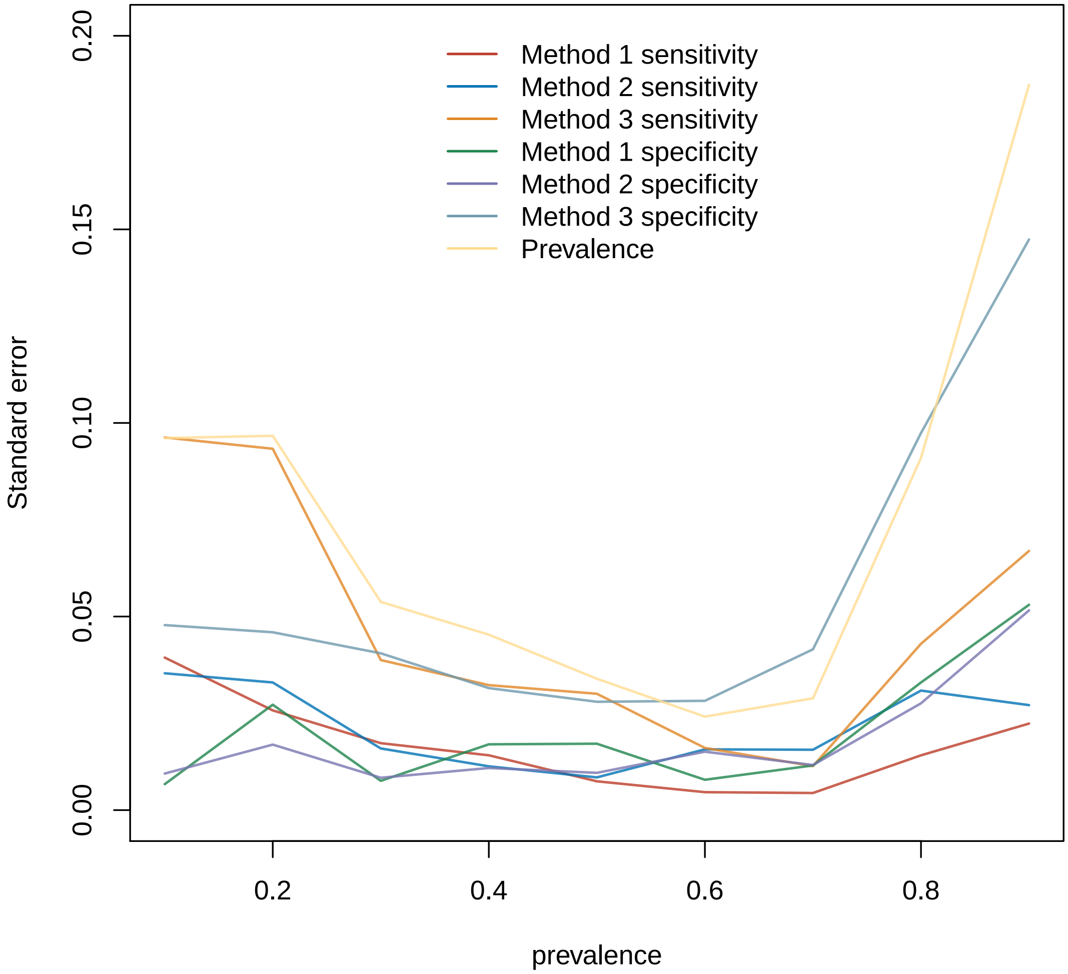

Figure S4: Relationship between simulated prevalence values and estimated standard errors. The simulated sample size was $100,000$. Each standard error was obtained from 99 times bootstrap resampling. The pre-defined true values of the operating characteristics were the same as in Figure 1.

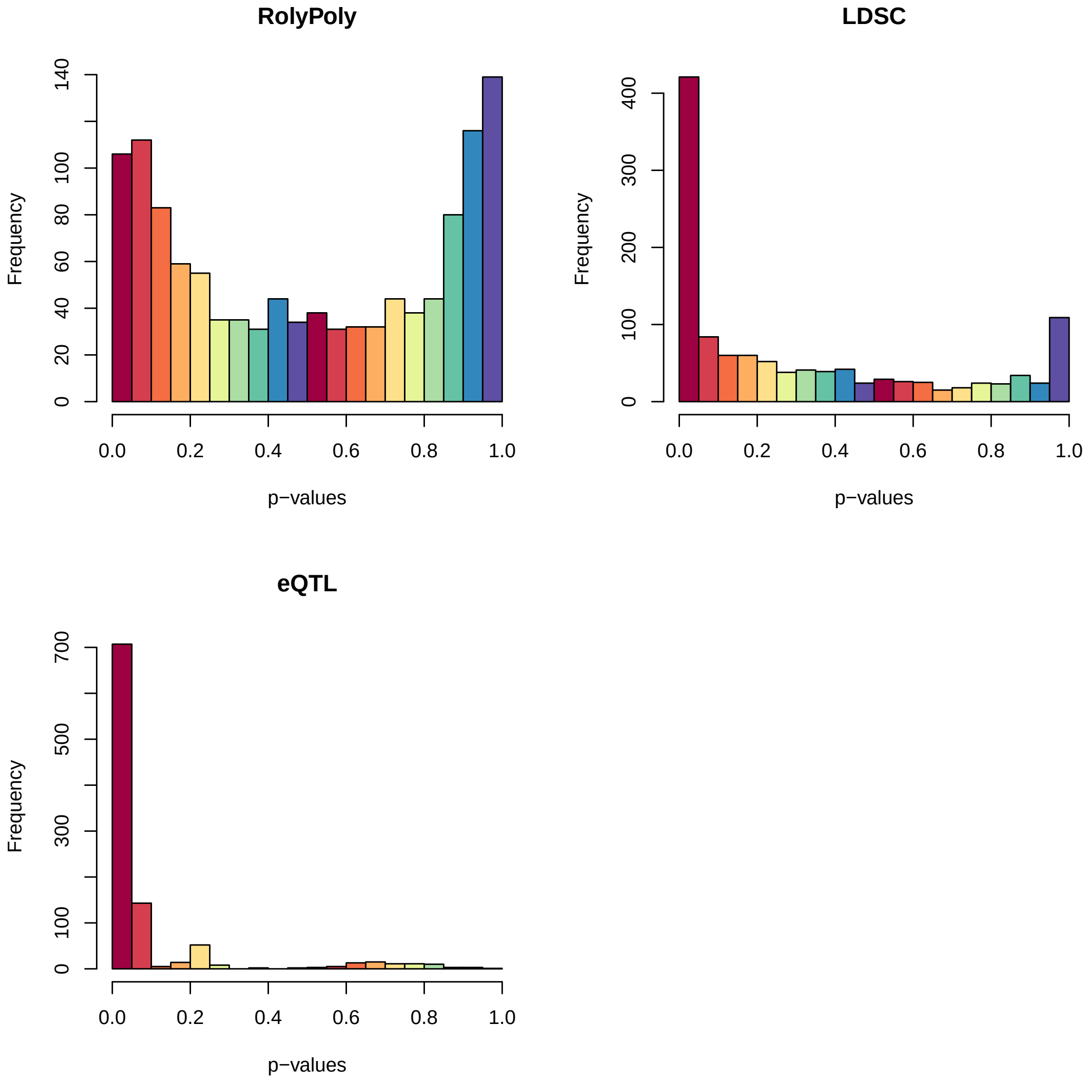

Figure S5: Tissue-trait association p-value distributions of the three methods. Each distribution was obtained from the analysis of 44 tissues and 27 complex traits.

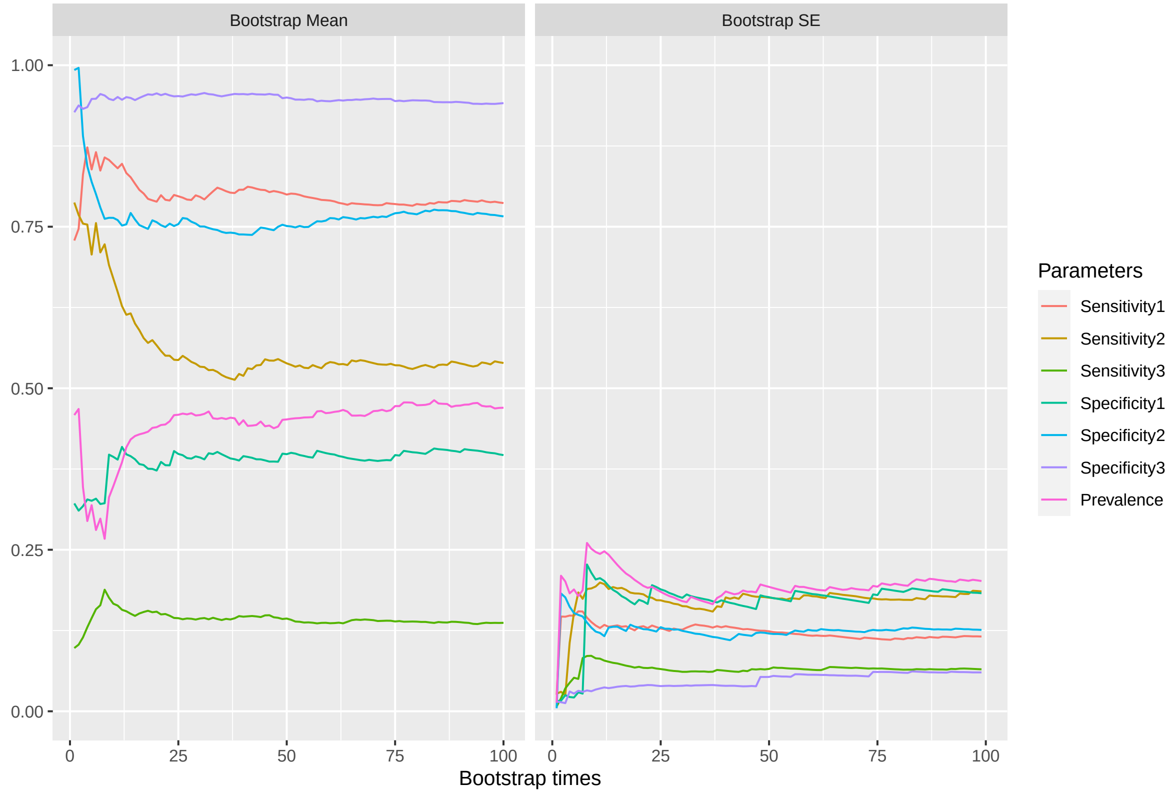

Figure S6: Example of the convergence of the bootstrap mean and standard error estimates for the parameters. The bootstrap results were produced under 0.05 p-value threshold for each method. Method 1, 2, and 3 stand for eQTL, LDSC, and RolyPoly, respectively.

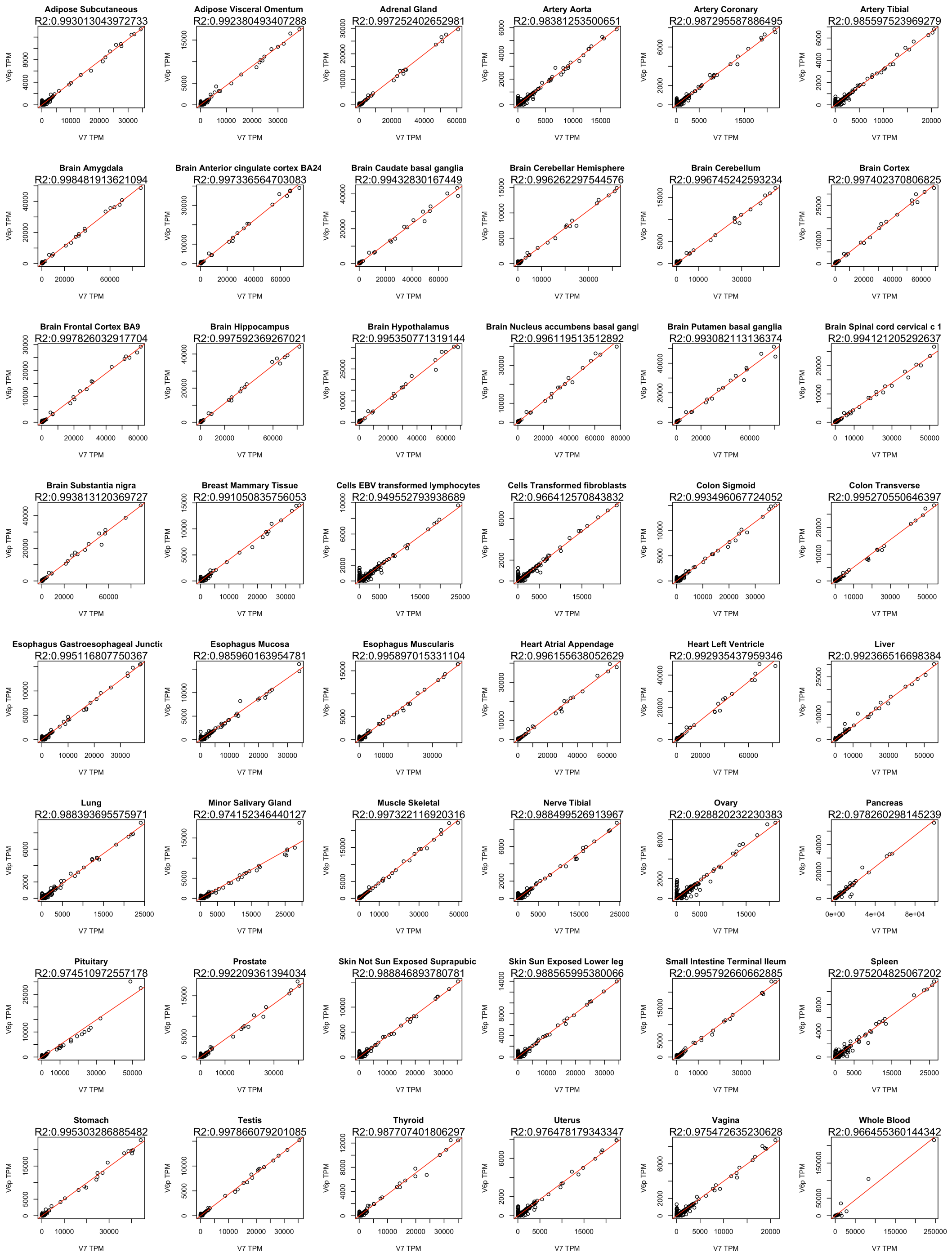

Figure S7: Comparison of the median gene expression values between GTEx version 6p and 7. The red lines represent the fitted simple linear regressions, and R2 are the corresponding R-squared values (coefficient of determination).
